# MRCQuant- an accurate LC-MS relative isotopic quantification algorithm on TOF instruments

**DOI:** 10.1186/1471-2105-12-74

**Published:** 2011-03-15

**Authors:** William E Haskins, Konstantinos Petritis, Jianqiu Zhang

**Affiliations:** 1 Pediatric Biochemistry Laboratory, University of Texas at San Antonio, TX, 78249, USA; 2 Depts. Biology & Chemistry, University of Texas at San Antonio, TX, 78249, USA; 3 RCMI Proteomics & Protein Biomarkers Cores, University of Texas at San Antonio, San Antonio, TX 78249, USA; 4 Dept. of Medicine, Division of Hematology & Medical Oncology, Cancer Therapy & Research Center,University of Texas Health Science Center at San Antonio, San Antonio, TX, 78229, USA; 5 Center for Proteomics, Translational Genomics Research Institute, Phoenix, AZ 85004, USA; 6 Dept. Electrical and Computer Engineering, University of Texas at San Antonio, TX 78249, USA

## Abstract

**Background:**

Relative isotope abundance quantification, which can be used for peptide identification and differential peptide quantification, plays an important role in liquid chromatography-mass spectrometry (LC-MS)-based proteomics. However, several major issues exist in the relative isotopic quantification of peptides on time-of-flight (TOF) instruments: LC peak boundary detection, thermal noise suppression, interference removal and mass drift correction. We propose to use the Maximum Ratio Combining (MRC) method to extract MS signal templates for interference detection/removal and LC peak boundary detection. In our method, MRCQuant, MS templates are extracted directly from experimental values, and the mass drift in each LC-MS run is automatically captured and compensated. We compared the quantification accuracy of MRCQuant to that of another representative LC-MS quantification algorithm (msInspect) using datasets downloaded from a public data repository.

**Results:**

MRCQuant showed significant improvement in the number of accurately quantified peptides.

**Conclusions:**

MRCQuant effectively addresses major issues in the relative quantification of LC-MS-based proteomics data, and it provides improved performance in the quantification of low abundance peptides.

## Background

The large-scale identification, characterization and quantification of proteins in biological samples by liquid chromatography-mass spectrometry (LC-MS) and liquid chromatography-tandem mass spectrometry (LC-MS/MS)-based proteomic methods play a crucial role in biomedical research [1,2]. For example, in biomarker discovery studies, a common aim is to elucidate a set of proteins that can be used to reliably differentiate diseased and normal samples by abundance measurements. Precision and accuracy are critical for confident protein biomarker discovery and validation. In "bottom-up" approaches, proteins are cleaved by sequence-specific proteases such as trypsin prior to analysis. A protein fold change can be inferred from the relative abundance of peptides across samples, where peptide identification and quantification can be accomplished in separate steps [3]. In this paper, we consider the problem of relative isotopic quantification of peptides in LC-MS based on time-of-flight (TOF) instruments. It is assumed herein that a list of candidate peptides has been compiled *a priori*, and that we are interested in measuring the relative abundance of their isotopes (natural or labeled).

The measurement of peptide abundance is complicated by the fact that a peptide forms both LC and MS peaks during its LC elution interval. To quantify a peptide, it requires the integration of its complete LC peaks, which is sometimes impossible due to strong interference from other peptide species or contaminants. However, relative quantification is still possible for the uncorrupted segments of LC peaks with slightly different isotopic compositions. Relative isotope abundance measurement is particularly important in chemical and metabolic labeling experiments for the quantification of differential expression of isotopically-labeled peptide pairs and their corresponding proteins. In "label-free" LC-MS peptide detection, measurement of relative natural isotope abundance is employed for peptide detection. In both cases, there exist several significant challenges: 1. The determination of LC peak boundaries to exclude noisy scans; 2. Background noise suppression in LC peaks; 3. Interference detection and removal; and 4. Mass drift correction. To achieve accurate relative quantification, these issues have to be addressed. Current software packages have not addressed these issues effectively. QUIL [4] and ProteinQuant [5] determine LC peak boundaries by the apex and the full-width-half-maximum (FWHM) of a peak, i.e., it is assumed that for a given LC elution peak, the distance between its starting point and its apex is the FWHM of the peak. This assumption is problematic when elution peaks (especially low abundance ones) are asymmetrical and jagged. Some software packages use an intensity threshold or local minima to determine the boundaries of LC peaks. The main problem of these methods is: one is never sure whether noise or interference-corrupted scans are included within the peak boundaries, which could greatly degrade quantification accuracy. Among popular software packages, msInspect [6] and SuperHirn [7] use thresholds, ASAPRatio [8] and MapQuant [9] use peak apex and FWHM. Recently, MaxQuant [10] uses local minima for LC peak detection after Extracted-Ion-Chromatogram (XIC) smoothing. See [2] for a comprehensive review of software tools currently available for LC-MS quantification.

On the problem of background noise suppression, almost all current software packages use Savitzky-Golay or other types of filters [6,8,10] to smooth XICs. However, through our own observation, elution process variations share similar frequency characteristics with that of instrument and Poisson noise (see [Additional file 1] for a detailed description of this phenomenon). Applying filters will distort elution process variations which adversely affect quantification accuracy.

For interference detection and removal, most software packages de-convolute peptide peaks and only consider peak centroids. Although this procedure decouples peptides with similar masses to a degree, it is susceptible to thermal noise, which can cause errors in the calculation of peak centroids. In addition, this procedure cannot provide interference detection, which is critical for accurate quantification.

Also, automatic mass drift correction is not implemented in these software packages, and users are generally expected to supply mass calibration information. This requirement introduces another source of variability, since the accurate determination of mass drift over all *m*/*z *ranges is a challenging problem. These issues become more severe when peptide abundance is low. Consequently, they have been bottlenecks in quantitative proteomic studies. For example, it is observed that whenever the signal intensity is low, the measurement of isotopically-labeled peptide pairs tends to be erroneous [11]. If we can computationally improve the coverage of accurate quantification, the chance for protein biomarker discovery will improve accordingly.

We limit the scope of this paper to TOFMS instruments where the Gaussian additive thermal noise model is appropriate [12,13], (note that this is different from the Poisson plus multinomial noise model for the XICs). In contrast, in FTMS, the assumption of Gaussian additive noise does not hold which is noted in [12] as the phenomenon of increased noise in XICs.

In this paper, we propose a Maximum Ratio Combining (MRC) based Quantification (MRCQuant) algorithm to address current issues in quantification. MRCQuant was developed based on the observation that peptide species register identical MS peak signals (scaled and noise corrupted) in different MS scans and *m/z *locations. Sometimes, the registered peaks have high Signal-to-Noise ratios (SNRs), while in other occasions, the peaks are noisy with low SNRs. While quantification at high SNRs is very accurate, quantification at low SNRs is problematic due to noise. We can extract the Maximum Likelihood estimate of peptide MS signals from MS peaks at high SNRs using MRC, hence referred to as *MS templates*. Note that these templates are extracted directly from experiment, and are not "predefined", thus they can capture slight variations in the shape and center locations of MS peaks caused by different environmental factors and instrument designs. Subsequently, extracted MS templates can be used as references when quantifying low SNR peaks. This method can effectively remove background noise without filtering out elution process variations. In addition, extracted MS templates can be compared to MS peaks for interference detection and removal. After interference and noise removal, accurate quantification can be performed.

MRCQuant provides measurements of isotopic abundance for each peptide of interest at all charge states and all isotope positions of interest. The output of the algorithm can be further processed to infer relative protein abundance in labeled experiments, or the results can be used for peptide detection based on isotope pattern in LC-MS data. The peptide list of interest can be compiled from peptides identified from multiple LC-MS/MS runs or from LC-MS peak detection algorithms such as msInspect [6].

### Definitions

Before we describe the MRCQuant algorithm, we first define several key terminologies that we use throughout the paper.

1. Maximum Ratio Combining (MRC) is an averaging method that has been widely applied in Telecommunications [14] for estimating the actual transmitted signal from multiple copies received through Additive White Gaussian Noise (AWGN) channels. MRC assigns averaging weights proportional to the square root of SNRs of received copies. MRC is mathematically derived based on the Maximum Likelihood principle. MRC provides an estimation of the transmitted signal with the highest SNR possible among all averaging methods. Given a peptide, we consider its MS peaks in multiple MS scans as copies of its real MS signal, which can be optimally estimated through MRC.

2. A reference template, not specific to any particular peptide, is defined as an estimation of the general MS peak shape in an LC-MS experiment. Such a peak shape is usually determined by instrument characteristics and environmental factors. Slight variations could exist between a reference template and particular peptide peak. This template can be translated and adjusted (in width) to different mass/charge (*m/z*) locations. (See support information for details of template translation). A reference template is described by its center *m/z *and its *m/z *-intensity pair values. Reference templates can either be extracted from LC-MS datasets at high SNRs, or can be theoretically predicted based on instrument resolution and characteristics. There may be several reference templates at different *m/z *values in an LC-MS dataset.

3. A peptide template is defined as an estimation of the MS peak signal registered by a specific peptide in one experiment. Comparing to reference templates, peptide templates are better estimations of MS peak signals for individual peptides. Peptide templates are generally extracted from MS peaks registered at the highest (most abundant) isotope and charge state position of peptides, where SNRs are high. Each peptide has its own template.

## Methods

### MRCQuant algorithm

Here we describe the MRCQuant algorithm for relative peptide isotope quantification on LC-MS. The input of the algorithm includes an LC-MS dataset and a list of peptides to be quantified annotated by their monoisotopic mass and/or amino acid sequence. The mass annotation can be obtained through an LC-MS peptide identification algorithm like msInspect. The output of the algorithm is a matrix of abundance measurements, with a maximum of *P *columns, where *P *is the total number of peptides to be quantified, and whose rows are indexed by cs _* _maxcs + *iso*, where *cs *∈ [1*, maxcs*] represents charge state, *maxcs *is the maximum number of charge states considered, and *iso *represents the isotope position. For a given peptide, we need to first detect its LC peaks. A peptide at a given mass forms a series of 2 D peptide peaks at different isotope and charge state positions. These 2 D peaks form LC and MS peaks if they are viewed from the elution time and *m*/*z *dimension. To establish the connection between a group of 2 D peaks to a specific peptide mass, we need to verify that: 1. their LC peaks at different isotope and charge state positions should be the same; and 2. their MS peaks match a reference template translated to their expected *m*/*z *locations. After LC peak identification, we need to accurately detect LC peak boundaries and perform quantification. To accomplish these goals, the proposed algorithm performs the following: 1. Extracts or theoretically predicts reference templates. 2. For each peptide of interest, performs LC peak detection at its highest isotope and charge state position using a reference template. 3.

Extracts peptide templates based on the MRC principle, which are used for accurate LC peak boundary detection and interference/noise removal at lower SNRs. Finally, quantification is performed based on peptide templates. The goal of the algorithm is to record accurate relative ion counts at all charge states and isotope positions.

**Figure 1:**
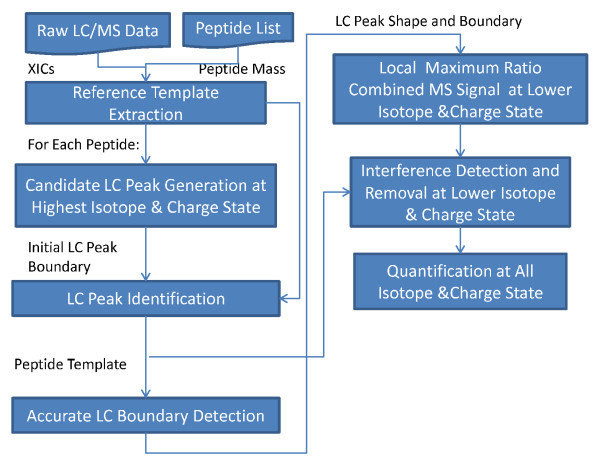
**Flow diagram of the MRCQuant algorithm**.

A flow diagram of the entire process is shown in Figure 1, which is explained in detail below.

### Generation of reference templates

Reference templates can either be extracted from experiments directly, or obtained through theoretical prediction. Theoretically predicted templates can adopt different peak shapes according to different instrument characteristics (resolution for example). Mass drifts can be accounted by shifting the center of theoretically predicted templates according to mass calibrations. Next, we discuss in detail how to extract reference templates from LC-MS data at high SNRs. Given an input peptide list, we select a subset of peptide that register uncorrupted MS peaks, from which we extract a number of reference templates centered at different *m/z *values. The underlying assumption is that MS peaks registered by the same instrument should be similar (except that MS peaks are scaled both in *m/z *and intensity). Thus, it is possible to use the estimated MS peak signals at high SNRs as reference templates for initial LC peak detection. Note that slight deviation of actual MS signals to reference templates is allowed since the templates are used for LC peak detection but not quantification. The number of templates can be selected by the user, and 4 templates have been used in our simulations with good results. Later, when quantifying a peptide at a given *m/z *value, we will not use a reference template, instead, we will use a peptide template for accurate quantification at low SNRs. This ensures that the template with the closest *m/z *value will be selected for LC peak boundary detection, interference detection and removal.

To extract the list of reference templates, we go through the following process for each peptide in the input list:

1. Determine the XICs of the peptide of interest at all charge states and isotope positions.

2. Determine the LC elution interval for the peptide of interest. To achieve this, we apply a high threshold at half maximum of the most intense (base) LC peak among all XICs. On the XIC with the tallest LC peak, all intervals above the threshold are considered as possible LC elution intervals. Then at the charge state of the base peak, we further check the correlations between the LC peaks on possible intervals at the two highest isotope positions (usually ^12^*C *and ^13^*C*). The interval corresponding to the peptide of interest should have a high correlation; otherwise the LC peaks must have been registered by other peptides, or have been corrupted by interference signals. The correlation is checked by R-squared statistics [15], and we apply a stringent threshold (> 0.9). We accept LC intervals with correlations higher than the threshold. If none of the intervals pass the threshold, we move on to the next peptide for possible template extraction. If multiple intervals have high correlations, which indicates that multiple peptides with similar mass occur on the same XIC, then we reject all intervals and move on to the next peptide since we can not detect the peptide interval unambiguously. This iterative procedure ensures that 1. We select a correct and unambiguous elution interval for the peptide of interest, and 2. The MS signal has not been corrupted by interference or noise.

3. If the elution interval is accepted, we determine the range of *m/z *values that the reference template spans (defined as the MS window of the template). The size of the MS window is determined by instrument resolution.

4. We average all MS peaks within the MS window and the accepted elution interval based on the MRC principle. The resulted MRC signal is an estimation of the MS peak signal registered by the peptide, and it can be used as a reference template.

After performing the above steps for each peptide, a list of reference templates has been obtained for LC peak detection. The details of XIC extraction, determination of MS windows, and the theoretical derivation of reference templates can be found in [Additional file 1].

### LC peak detection

After obtaining a list of reference templates, the algorithm moves on to accurately detect and quantify the LC peak for each peptide of interest. Given a peptide, we start LC peak detection by inspecting its XICs. Usually, several LC peaks above the background noise level exist on an XIC, where, one is generated by the peptide of interest and the rest belong to others. We need to correctly identify the LC peak and its boundaries so that noise signals are not included in relative quantification. We perform the following processing steps:

#### Candidate LC peak generation

The goal of this step is to detect high intensity intervals (LC peak candidates) on XICs of the peptide of interest for further investigation. Ideally, we should perform such detection at the most abundant charge state and isotope position where the LC peak has the highest SNR possible. Given peptide sequence information or mass, it is possible to predict its isotopic pattern [16], and its most abundant isotope position (base position). On the other hand, it is difficult to predict the most abundant charge state, and an exhaustive search must be conducted. We perform the following processing steps at all charge states:

1. Given a peptide's mass (m) at a charge state (z), determine its theoretical *m/z *values at different isotope positions.

2. At the *m/z *value of its base peak, estimate its MS window and generate the XIC.

3. Apply an intensity threshold at 3 times the estimated background noise standard deviation to identify LC peak candidates.

4. Determine the FWHM elution intervals of LC peak candidates by applying thresholds at half maximum of these LC peak candidates. These FWHM boundaries are set as initial LC peak boundaries. In this way, we only include MS scans with relatively high SNRs.

5. Check the correlation between LC peaks at the most intense and the second most intense isotope positions within the initial boundaries of each LC peak candidate. The correlation is checked using R-squared statistics, and all candidates with R statistics greater than 0.9 will be accepted. In this way, all intervals with good correlations between two isotopes will be selected.

6. If the maximum R-statistic is less than 0.9, then the LC peak candidate with the maximum R statistics will be selected. This corresponds to the case when correlations between isotope elution profiles are poor due to noise or interference, and the peptide of interest may or may not exist. In such cases, we select the best candidate for further verification in the MS dimension.

At the end of this process, a list of *k *LC peak candidates, each denoted by its start and end scan, is generated at each charge state. The charge state with the highest total ion count within initial LC peak boundaries will be selected.

Next, one of these LC peak candidates will be identified as the initial LC peak.

#### LC peak identification

From previous steps, we find *k *LC peak candidates, but generally only one of them is generated by the peptide of interest, which can be further identified by matching a reference template to the MS peaks within the elution interval of each candidate:

1. We select the closest reference template to the peptide of interest in *m/z *values, which ensures the best match between the template and local MS peaks. We then translate the template to the local *m/z *value of the peptide of interest. Details of template translation can be found in [Additional file 1].

2. For each LC peak candidate, estimate its local MS peak signal by averaging all MS peaks (using MRC) within its initial boundaries. By employing MRC, noise in individual MS peaks will be maximally suppressed.

3. The estimated local MS signal will be compared to the selected reference template. The LC peak candidate with the best matched local MS signal will be identified as the final LC peak.

4. If none of the local MS signals match with the reference template well (with R statistics < 0.4), then LC peak detection failed for the given peptide. This could happen when a peptide identification algorithm wrongly reports the center mass of the peptide, which leads to a mismatch between the reference template and the local MS signal. Although it is possible to correct such wrongly reported mass, however, it is beyond the scope of this paper.

At the end of this processing step, an LC peak has been identified for the peptide of interest with initial boundaries detected using a high intensity threshold at half the maximum of the LC peak.

We do not assume specific LC peak shapes (e.g. Gaussian), and the algorithm can be applied in various LC conditions (e.g., different reverse-phase gradients). If reference templates are extracted from an LC-MS experiment directly, then they will be centered at their theoretical *m/z *values plus the mass drift of the experiment. Thus, mass drift will be automatically accounted when applying such reference templates for LC peak detection. If a theoretical reference template is used, then its center needs to be shifted according to user provided mass calibration information.

#### Peptide template extraction

For a peptide of interest, its identified LC peak has an initial elution interval that covers the intensity region above half of the LC peak maximum, and it is obtained at the highest charge state and isotope position. These conditions ensure that the MRC signal associated with the identified LC peak is estimated at a high SNR, and it can be treated as the peptide template of interest. Such a template captures accurate MS peak shape information, which can be used for LC peak boundary detection and quantification.

#### Accurate LC peak boundary detection

The initial LC peak boundaries are obtained by applying a high intensity threshold, and many MS scans that belong to the peptide of interest are excluded. We need to accurately extend the boundaries so that all MS scans of the peptide will be accounted. If the boundaries exclude a significant segment of the LC peak, then quantification will be less accurate since combining fewer scans cannot suppress noise sufficiently. If the boundaries are extended too far to include scans that contain interference and noise, then quantification accuracy will also be reduced.

The problem of LC peak boundary detection can be translated to the problem of detecting of all scans that contain the peptide template. It can be further formulated as a hypothesis testing problem:

H0: A given MS scan only contains noise;

H1: The scan contains noise plus the peptide template.

We test the hypothesis by comparing the peptide template to the MS peak signal in a given scan. If the R-statistic is greater than a threshold (0.5), then H1 is accepted.

We start this hypothesis testing procedure from the initial LC peak starting scan to extend the head of the LC peak. Then we apply the same procedure to the tail end of the peak. Whenever encountering a scan that does not contain the template, the extension process will be terminated.

**Figure 2:**
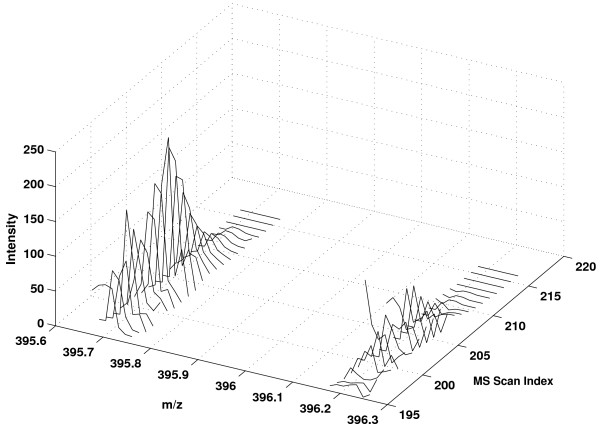
**Example of peptide 2 D peaks with interference at *C*^12^**. Comparison of *C*^12 ^and *C*^13 ^peaks reveals interference at *C*^12 ^in scans 194 - 199.

**Figure 3:**
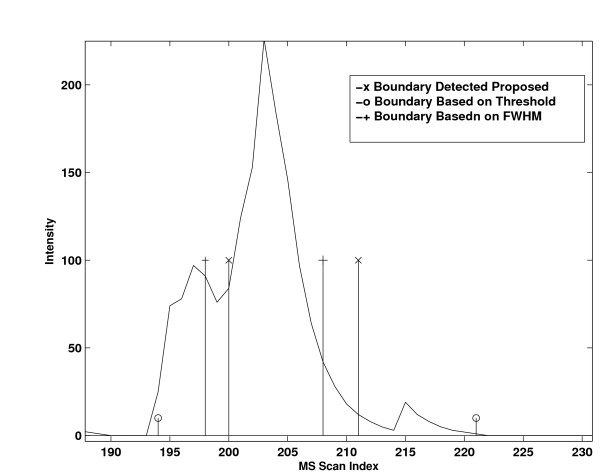
**Comparison of different boundary detection methods**. Comparison of *C*^12 ^and *C*^13 ^peaks reveals interference at *C*^12 ^in scans 194 - 199.

Accurate boundary detection plays a critical role in quantification accuracy. For example, in Figure 2, we plot the 2 D peaks of a peptide at ^12^*C *and ^13^*C *positions in charge state 2. The peptide signal actually resides from scan 200 to 211. In scan 194 - 199, an interfering peptide with similar m/z produces MS peaks at the ^12^*C *position. However, inspecting the peaks at ^13^*C*, it is evident that interference peaks do not exist in scans 194 - 199. If the interfering scans are included, the resulted relative quantification accuracy will be greatly degraded. In Figure 3, we compare different boundary detection methods. The threshold method includes all scans from the interfering peptide. The FWHM method includes a few interfering peptide scans and excludes a few scans that belong to the peptide of interest. In contrast, the proposed method accurately detected the boundary from scan 200-211.

### Quantification

For a given peptide, we have obtained its peptide template and LC peak boundaries after LC peak detection. Based on these inputs, we can accurately quantify the peptide at other charge state and isotope positions. At a given "local *m/z *value" of low SNR, quantification consists of three processing steps: 1. Obtain a local MRC signal by averaging all MS peaks (using MRC) within the detected LC peak boundaries to optimally suppress noise; 2. Compare the translated peptide template with the local MRC signal for interference detection and removal. This step also provides an estimation of the scaling factor for the local MRC signal in reference to the peptide template, which can be multiplied to the total ion count of the template to derive the total ion count of the peptide at the local *m/z *value.

Local MRC signal are derived using weights proportional to the LC peak intensities obtained at the LC peak detection stage. The details of other processing steps are described below.

#### Interference detection and removal

The input to this processing step includes the local MRC signal and the translated peptide template, whose correlation is calculated using the R-square statistic [15]. If the correlation is greater than 0.9, then it is considered that the interference signal does not exists. Otherwise, the local MRC signal is considered as interference corrupted, and we have to perform interference removal within its MS window.

We model a local MRC signal as the superposition of the translated peptide template (scaled by *a*) and an interference signal which is modeled as an order *l *polynomial. The interference removal problem is equivalent to the accurate estimation of the scale factor *a *and the polynomial parameters.

When assuming Gaussian noise, the Maximum Likelihood estimation of these parameters is equivalent to their least-square-estimation (LSE). Note that the correlation between the interference and the peptide template signal must be minimized to yield a good estimate of *a*. Otherwise, the estimated interference signal could contain partial template signal. Consequently, besides finding the LSE of parameters, the second objective is to find parameters that minimize the correlation between the template and the interference signal. In addition, there is the constraint that both the template and the interference signal should be positive at all *m/z *values. These requirements lead us to formulate a constrained multiple objective optimization problem. We utilize the Quadratic Programming algorithm [17] to numerically search for the solution of model parameters. The selection of polynomial order is based on the Bayesian Information Criteria (BIC) [18]. See [Additional file 1] for details.

**Figure 4:**
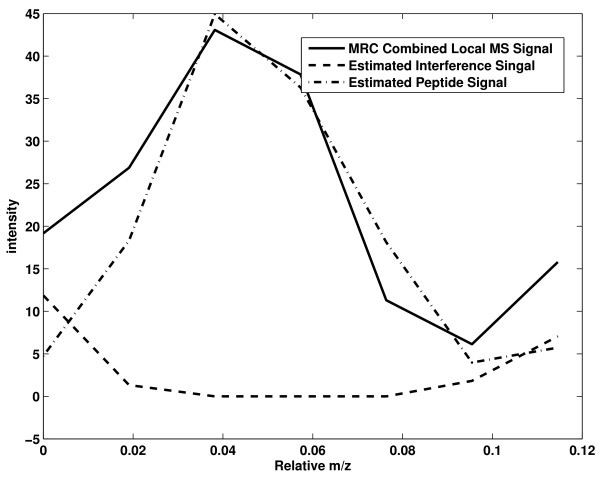
**Interference Removal**. The MS template is extracted at a high SNR. The local MRC MS signal is derived at a lower SNR. The local MRC MS signal deviates from the extracted MS template significantly.

Figure 4 shows an example of interference removal. The peptide template in Figure 4 is extracted at a high SNR. The local MRC signal is derived at a lower SNR. Due to interference, the local MRC signal deviates from the peptide template significantly. We employ the proposed interference removal method to estimate the interference and peptide signal. When performing quantification, the interference signal is not counted towards the total ion count.

Note that there exist various peak identification algorithms [19,20] that are specially designed to deal with the problem of overlapping peptide peaks. These algorithms are generally exponentially complex with the number of overlapping peaks considered. In this paper, the focus is on accurate quantification after peptide identification. Thus, the problem is simplified to only extract signals for the peptide of interest. The knowledge of overlapping peptides can help in improving quantification accuracy, but since peak identification algorithms may or may not provide such information, we uniformly treat overlapping signals as interference. The MRC process also has the effect of suppressing interfering signals since higher weights are given to tall MS peaks of the peptide of interest but not interfering peaks. This treatment also limits the computational complexity, which is linear to the number of peptides to be quantified.

#### Quantification based on local MRC signal

At the end of interference removal, the local MRC signal is cleaned of interference and the scale factor *a *is also derived. It is easy to show that the total ion count *C_s _*of all MS peaks within the LC peak interval and the total ion count of the local MRC MS signal *C_m _*has the relationship(1)

where *w*(*t*) are normalizing weights used for MRC. Thus if the total ion count of the peptide template is *C_t_*, the total ion count of the LC peak *C_p _*can be estimated as , where C_t _* a = *C_m _*is the estimated total ion count of the local MRC signal.

**Figure 5:**
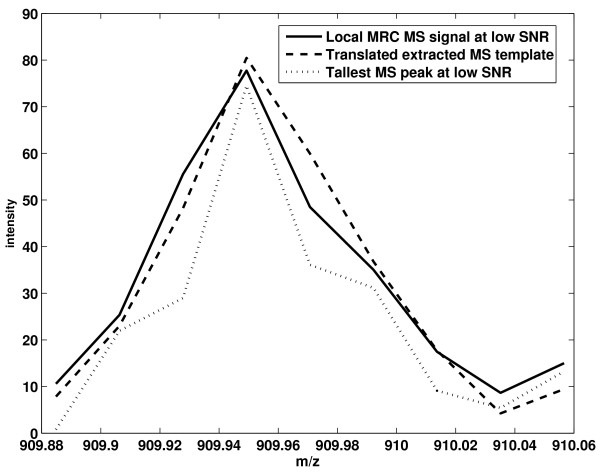
**The Translated template at a lower isotope position**. The peptide signal in an individual scan is very noisy (signal in dashed line). In contrast, the MRC combined signal has a much higher SNR, and it is very close to the extracted MS template.

In Figure 5, we show an example of the effect of noise reduction by MRC. At a lower SNR position, the peptide signal in an individual scan is very noisy (signal in dashed line). In contrast, the local MRC combined signal has a much higher SNR, and it is very close to the peptide template.

### Data Collection and processing

We developed our algorithm based on an LC-MS dataset collected from a tryptic digest of horse myoglobin at a concentration of 600 fmol (unless noted, all illustrations in this paper are generated based on this dataset). For reference, we also obtained an LC-MS/MS dataset for peptide sequence information at 100 fmol. LC-MS/MS was performed with a splitless nanoLC-2 D pump (Eksigent), a 50 *μ*m-i.d. column packed with 10 cm of 5 *μ*m-o.d. C18 particles, and a linear ion trap tandem mass spectrometer (LTQ-XLS; ThermoFisher). The top 7 most abundant eluting ions were fragmented by (data-dependent) collision-induced dissociation (CID). The LC gradient was 2 to 98% 0.1% formic acid/acetonitrile in 60 min (60-120 min) at 400 nL/min. Tandem mass spectra were extracted by Mascot Distiller version 2.3.1. Charge-state-deconvolution and deisotoping were not performed. All MS/MS samples were analyzed using Mascot (Matrix Science, London, UK; version 2.3.2). Mascot was set up to search the Swiss-Prot database assuming the digestion enzyme trypsin. Mascot was searched with a fragment ion mass tolerance of 0.80 Da and a parent ion tolerance of 2.0 Da. Oxidation of methionine and iodoacetamide derivative of cysteine were specified in Mascot as variable modifications. LC-MS was performed with a splitless nanoLC-2 D pump (Eksigent), a 50 *μ*m-i.d. column packed with 10 cm of 5 micro-o.d. C18 particles, and a time-of-flight mass spectrometer (MicrOTOF; Bruker Daltonics). The LC gradient was 2 to 98% 0.1%formic acid/acetonitrile in 60 min (60-120 min) at 400 nL/min. Mascot search correctly linked 13 peptides observed in the sample to horse myoglobin with an 80% sequence coverage.

For algorithm verification, we downloaded a QTOF dataset from the repository of Seattle Proteome Center at http://regis-web.systemsbiology.net/PublicDatasets/. The repository was created for testing various algorithms. It contains LC-MS/MS datasets of an 18 protein digest. For details of data collection please refer to [21]. There are multiple LC-MS/MS datasets collected on various instruments within the repository. We downloaded datasets related to protein mixture 4 of the 18 protein mix. Among which, from a total of 21 runs on LTQ-FT, QStar and QTOF, we compiled a list of 784 LC-MS/MS-identified peptides for the same protein mixture. These peptides were all identified with a PeptideProphet™[22] score greater than 0:9. We also performed LC-MS peak detection using msInspect on one of the QTOF datasets *QT *20060925_*mix*4_23.mzxml (mix4_23) that identified 1952 peptides. Subsequently we quantify these peptides by MRCQuant. MsInspect was selected because it is the most representative LC-MS peptide identification and quantification algorithm and has been shown to outperform other peak detection algorithms [23]. It applies a conservative noise threshold initially. Subsequently, MS scans are centroided; XICs are smoothed; LC peak length filter is applied; and LC peaks that appear and disappear together are pooled and treated as signals registered by identical peptides at different isotope positions and charge states. Subsequently, peptides are identified by comparing their theoretically predicted isotope patterns and measured isotope patterns using Kullback-Leibler(KL) distance. Other popular software packages such as ASAPRatio [8] differ slightly in the details, but the main procedure, MS peak detection in each MS scan followed by quantification based on XICs, is similar to that of msInspect. Among these software packages, msInspect provides relative quantification accuracy measurements in the form of KL distance, which enables us to compare performances. Other software packages do not provide this measurements, therefore, relative quantification accuracy cannot be accessed.

When using the msInspect software package (Build 599) to process *mix*4_23 dataset, we tried to optimize the number of peptides being reported. We selected the "walksmooth" option when running the command "findPeptides", and we set msInspect parameters "minpeaks" to 2 and "maxkl" to 10. The "walksmooth" option greatly improves the number of features as well as the KL scores reported. A total of 1952 features were reported. In comparison, if the default settings of msInspect are used, 933 features were reported with worse KL scores.

The peptides reported by msInspect were further processed by MRCQuant. We used extracted templates at high SNRs as reference MS templates. We rejected some msInspect reported features either because: their reported msInspect KL scores are negative, or our algorithm determines that the LC peaks reported by msInspect cannot be found. The latter case could be caused by inaccurate mass reporting by msInspect. When the mass is reported inaccurately, the reference template and the local MS signal would deviate from each other significantly, and our algorithm rejects LC peaks when the R statistic between the reference template and the local MS signal is less than 0.4. Correcting the incorrectly reported mass is a peptide identification problem which is beyond the scope of this paper. This results in a peptide list of length 964 with accurately reported mass values.

### Relative quantification accuracy evaluation

To perform relative quantification accuracy evaluation, we need to introduce an appropriate metric. The ideal way to evaluate relative quantification accuracy is to compare the measured ratios of natural isotopes to that of theoretically predicted ones. However, none of the software packages report abundance levels at different isotope positions directly. MsInspect reports KL scores which can be used to access relative quantification accuracy indirectly. Given measured natural isotope ratios [*p*(1)*, p*(2), ⋯] and theoretically predicted ones [*q*(1)*, q*(2), ⋯], (When sequence information is available, natural isotope ratios can be calculated exactly. Otherwise, at a given mass, they can be estimated from its mass [24]), the KL score is evaluated using the following formula:(2)

If two sets of isotope ratios entirely agree with one another, then their KL score equals to zero. Otherwise, a KL score is always positive, and the larger it is, the bigger the difference between the two sets of isotope ratios.

Different KL scores indicate different levels of quantification accuracy, and it is possible to compare the performance of different algorithms by the reported number of peptides at different KL score thresholds. For example, we can claim that algorithm one is better than algorithm two, if algorithm one reports more peptides with KL scores less than a threshold.

Obviously, we cannot set the KL threshold to infinity, and now the question becomes what could constitute an "acceptable range of KL thresholds". We know that given a KL score, there always exist the probability that it is the divergence between an arbitrarily generated and an authentic isotope distribution. The higher the KL score is, the higher the probability. If the KL score of a reported peptide is high, it is very probable that the real peptide signal does not exist, and the reported isotope distribution is generated based on observations of random noise. This probability is defined as the False-Detection-Rate (FDR), which can be converted from a KL score in reference to a KL null distribution (the distribution of KL scores between authentic peptide and arbitrarily generated isotope ratios). Obviously, when the FDR is high, it is not meaningful to compare the reported number of peptides between two algorithms anymore, since a significant portion of reported peptides should have been falsely detected. In this paper, we adopt a cutoff FDR of 12%, and we compare the number of reported peptides at different FDRs less than 0.12.

Given a KL score reported by an algorithm, to convert it to FDR, the p-value of the KL score is first generated based on the KL null distribution. Subsequently, the FDR is estimated using the method described in [25] based on the p-value. The Matlab function, *mafdr*(·), is used to estimate the FDRs from the p-values.

The null distribution on KL score is generated by calculating the KL scores between arbitrarily generated isotope distributions with authentic ones. Without observations, an arbitrary distribution on isotopes is generated by drawing *maxiso *random numbers uniformly distributed on 0[1], and then these numbers are normalized to form a distribution. We generate authentic theoretical isotope distributions by randomly drawing mass values from the peptide list reported by msInspect, and then for these mass values, we calculate their theoretical isotope ratios using the method in [24].

## Results and Discussion

**Figure 6:**
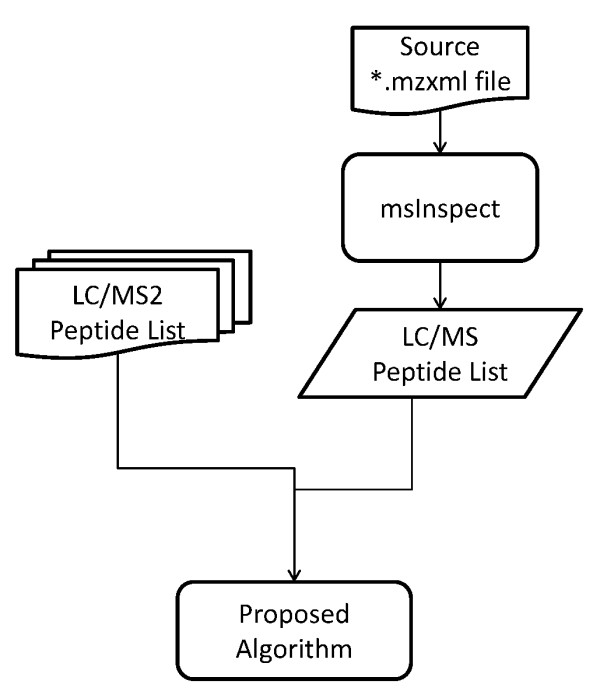
**Verification process**.

We applied MRCQuant to both peptide lists identified by msInspect and LC-MS/MS. The performance of MRCQuant is measured by the number of reported peptides at FDRs that are less then 0.12. Peptides reported with low FDRs/KLs are considered as accurately quantified ones. See Figure 6 for an illustration of the algorithm verification process. Note that the direct comparison of computing time between MRCQuant and msInspect is not possible because msInspect is a combined peak identification and quantification algorithm, while MRCQuant focuses on quantification only. The complexity of MRCQuant is linear in complexity, i.e. the processing time is linear to the number of peptides to be quantified. On a Dell T7500 workstation, the processing time for the msInspect list was below half an hour.

### Performance comparison between MRCQuant and msInspect

**Figure 7:**
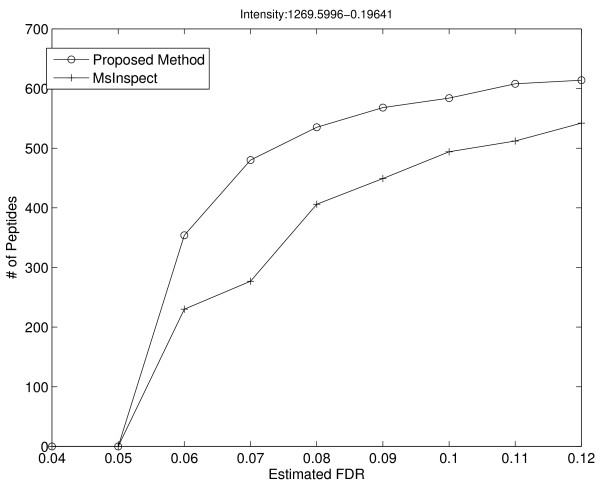
**Number of detections v.s. estimated FDR of the proposed algorithm and msInspect**. The proposed algorithm improves relative quantification accuracy greatly over msInspect on low FDR regions in the number of reported peptides.

We first compared the performance of MRCQuant to that of msInspect based on the msInspect reported peptide list. In Figure 7, we plot the number of reported peptides at different FDRs by MRCQuant and msInspect. From this figure, we can see that MRCQuant reports more accurately quantified peptides than msInspect at low FDRs. We used reference and peptide templates extracted from LC-MS data for these calculations.

We also compared the performance of msInspect and MRCQuant based on LC-MS/MS-identified peptides. However, when allowing a 10 ppm tolerance, there are only 31 LC-MS/MS-identified peptides that overlap with msInspect-reported peptides. In other words, most peptides compiled from multiple LC-MS/MS runs were not reported by msInspect. With such a small number of overlaps, we could not perform a meaningful comparison. In contrast to the low detection rate of LC-MS/MS-identified peptides by msInspect, MRCQuant quantified 423 LC-MS/MS-identified peptides in total, among which, 203 have an FDR <= 0.1.

### Performance at different intensity levels

**Figure 8:**
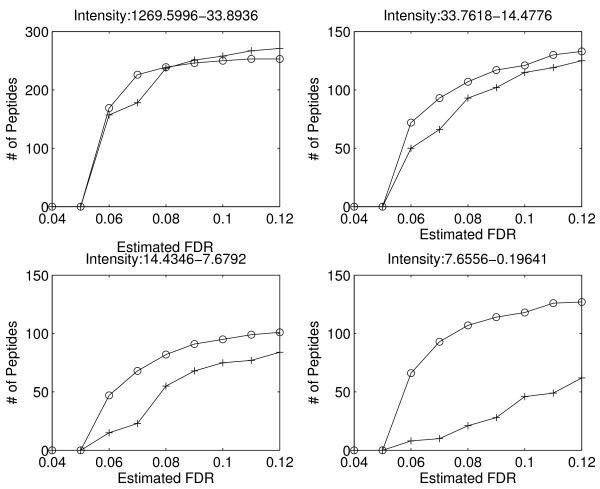
**Performance at different intensity levels**. As the intensity level lowers, the proposed algorithm provides better and better performance over msInspect in the number of reported peptides.

MRCQuant is mainly designed to correctly quantify peptides at low intensity levels where the effect of noise is the most detrimental. To evaluate the performance at different intensity levels, we sorted peptides according to their peak intensities reported by msInspect. Then, we divided peptides into 4 different groups according to their intensity levels, and we plotted the performance curves (the number of peptides v.s. FDR) as shown in Figure 8. MRCQuant clearly provides similar performance to msInspect in the high intensity region (33-1269); however, MRCQuant provides better performance over msInspect in lower intensity regions. Note that there are more peptides in low intensity regions (> 600) than in the high intensity region (300). Thus, MRCQuant has a much better performance on the low intensity regions where most peptides can be found.

### Effect of using different templates

**Figure 9:**
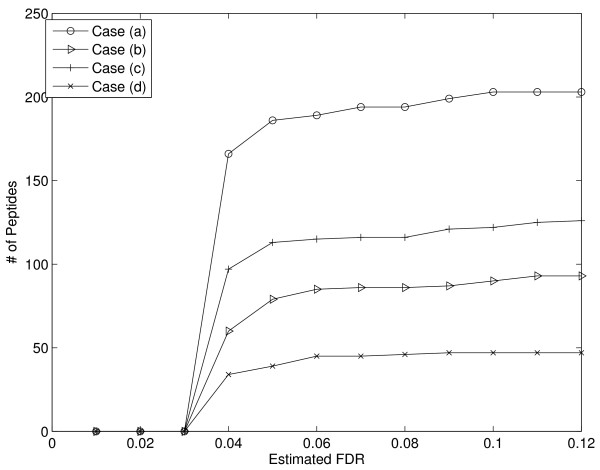
**Performance with different template selections**. (LC Peak Detection, Quantification): a. (Extracted Reference, Extracted Peptide); b. (Theoretical Reference, Extracted Peptide); c. (Extracted Reference, Extracted Reference); d.(Theoretical Reference, Theoretical Reference). The selection of templates greatly affects the quantification performance.

MRCQuant can be configured to use extracted or theoretically predicted reference templates for LC peak detection, and it can also be configured to use locally extracted peptide or reference templates for quantification. Thus, there are four possible ways of employing MRCQuant: (a). Use the extracted reference template for LC peak detection and use the extracted peptide template for quantification. (b). Use the theoretically predicted reference template for LC peak detection and use the extracted peptide template for quantification. (c). Use the extracted reference template for LC peak detection and quantification. (d). Use the theoretically predicted reference template for LC peak detection and quantification. We tested these four cases on the LC-MS/MS-identified peptide list. The performances are reported in Figure 9. The selection of templates greatly affects quantification performance. Case (a) uses the most accurate templates possible in both LC peak detection and quantification, and the result is the best with significantly higher number of reported peptides on the low FDR region. The comparison between case (a) and case (b) reveals the effect of mass drift on quantification accuracy. In case (a), the extracted reference templates are used, the mass drift in a specific LC-MS run is automatically addressed, and thus LC peak detection is more accurate. In case (b), the theoretically predicted MS reference template was not adjusted for mass drift and the resulted LC peak detection result is poor. Comparing case (a) and (c), we can see quantification accuracy degradation caused by not using extracted peptide templates. Slight variations in local signal peak shapes affect quantification accuracy significantly. We also compared the performance of using different templates based on msInspect generated peptide list. Again, more peptides are reported with low FRDs in case (a) than in other cases, which confirms the importance of using extracted templates.

## Conclusions

In this paper, we describe a new algorithm called "MRCQuant" for LC-MS relative quantification of "bottom-up" proteomics data based on extracted MS templates. Reference- and peptide- MS templates are extracted from scans with relatively high SNRs using MRC, a process that optimally suppresses noise.

Subsequently, these templates are used for detecting LC peak boundaries, detecting interference, and removing noise at lower SNRs. MRCQuant performs automatic mass drift correction by utilizing extracted MS templates which capture mass deviation from theoretical mass values. These techniques address major deficiencies in previous LC-MS quantification algorithms effectively. We demonstrate significant improvement in relative quantification accuracy with a larger number of detected peptides at low FDRs compared to msInspect. We expect that MRCQuant can be integrated with various LC-MS processing software to improve the overall performance. For example, MRCQuant can be readily modified and applied in label and label-free proteomic experiments for quantitative analysis. The proposed algorithm can also be incorporated in LC-MS peak detection algorithms that use isotope ratios.

## Authors' contributions

JZ conceived, developed, and implemented the algorithm. She also prepared the initial manuscript. WH performed the LC-MS/MS experiments and revised the manuscript. KP advised and revised the manuscript. All authors have read and approved the final manuscript.

## Availability and requirements

Relevant data and source Matlab scripts are available at **project home page: **http://compgenomics.utsa.edu/MRCquant.html

## Supplementary Material

Additional file 1 **Support Information**. In this file we provide support information. Click here for file

## References

[B1] Aebersold R Mann M Mass spectrometry-based proteomics Nature 2003 422 6928 198 207 10.1038/nature01511 12634793

[B2] Mueller L Brusniak M Mani D Aebersold R An assessment of software solutions for the analysis of mass spectrometry based quantitative proteomics data Journal of proteome research 2008 7 01 51 61 10.1021/pr700758r 18173218

[B3] Bantscheff M Schirle M Sweetman G Rick J Kuster B Quantitative mass spectrometry in proteomics: a critical review Analytical and bioanalytical chemistry 2007 389 4 1017 1031 10.1007/s00216-007-1486-6 17668192

[B4] Wang G Wu W Pisitkun T Hoffert J Knepper M Shen R Automated quantification tool for high-throughput proteomics using stable isotope labeling and LC-MSn Analytical chemistry 2006 78 16 5752 10.1021/ac060611v 16906720

[B5] Mann B Madera M Sheng Q Tang H Mechref Y Novotny M ProteinQuant Suite: a bundle of automated software tools for label-free quantitative proteomics Rapid Communications in Mass Spectrometry 2008 22 23 3823 3834 10.1002/rcm.3781 18985620

[B6] Bellew M Coram M Fitzgibbon M Igra M Randolph T Wang P May D Eng J Fang R Lin C A suite of algorithms for the comprehensive analysis of complex protein mixtures using high-resolution LC-MS Bioinformatics 2006 22 15 1902 10.1093/bioinformatics/btl276 16766559

[B7] Mueller L Rinner O Schmidt A Letarte S Bodenmiller B Brusniak M Vitek O Aebersold R Muller M SuperHirn-a novel tool for high resolution LC-MS-based peptide/protein profiling Proteomics 2007 7 19 3470 80 10.1002/pmic.200700057 17726677

[B8] Li X Zhang H Ranish J Aebersold R Automated Statistical Analysis of Protein Abundance Ratios from Data Generated by Stable-Isotope Dilution and Tandem Mass Spectrometry ANALYTICAL CHEMISTRY-WASHINGTON DC 2003 75 23 6648 6657 10.1021/ac034633i 14640741

[B9] Leptos K Sarracino D Jaffe J Krastins B Church G MapQuant: Open-source software for large-scale protein quantification Proteomics 2006 6 6 1770 1782 10.1002/pmic.200500201 16470651

[B10] Cox J Mann M MaxQuant enables high peptide identification rates, individualized ppb-range mass accuracies and proteome-wide protein quantification Nature biotechnology 2008 26 12 1367 1372 10.1038/nbt.1511 19029910

[B11] Ong S Mann M A practical recipe for stable isotope labeling by amino acids in cell culture (SILAC) Nature protocols 2007 1 6 2650 2660 10.1038/nprot.2006.427 17406521

[B12] Du P Stolovitzky G Horvatovich P Bischoff R Lim J Suits F A noise model for mass spectrometry based proteomics Bioinformatics 2008 24 8 1070 10.1093/bioinformatics/btn078 18353791

[B13] Shin H Koomen J Baggerly K Markey M Towards a noise model of MALDI TOF spectra American Association for Cancer Research (AACR) advances in proteomics in cancer research 2004

[B14] Goldsmith A Wireless communications 2005 Cambridge Univ Pr

[B15] Draper N Smith H Applied Regression Analysis 1998 ch. 10 3 Wiley-Interscience, New York

[B16] Bayne C Smith D A new method for estimating isotopic ratios from pulse-counting mass spectrometric data International Journal of Mass Spectrometry and Ion Processes 1984 59 3 315 323 10.1016/0168-1176(84)85105-8

[B17] Fletcher R Practical Methods of Optimization: Vol. 2: Constrained Optimization JOHN WILEY & SONS, INC., ONE WILEY DR., SOMERSET, N. J. 08873, 1981, 224 1981

[B18] Liddle A Information criteria for astrophysical model selection Monthly Notices of the Royal Astronomical Society: Letters 2007 377 L74 L78 10.1111/j.1745-3933.2007.00306.x

[B19] Renard B Kirchner M Steen H Steen J Hamprecht F NITPICK: peak identification for mass spectrometry data BMC bioinformatics 2008 9 355 10.1186/1471-2105-9-355 18755032 PMC2655099

[B20] Wang Y Zhou X Wang H Li K Yao L Wong S Reversible jump MCMC approach for peak identification for stroke SELDI mass spectrometry using mixture model Bioinformatics 2008 24 13 i407 10.1093/bioinformatics/btn143 18586741 PMC2718621

[B21] Klimek J Eddes J Hohmann L Jackson J Peterson A Letarte S Gafken P Katz J Mallick P Lee H The standard protein mix database: A diverse dataset to assist in the production of improved peptide and protein identification software tools Journal of proteome research 2008 7 96 10.1021/pr070244j 17711323 PMC2577160

[B22] Keller A Nesvizhskii A Kolker E Aebersold R Empirical statistical model to estimate the accuracy of peptide identifications made by MS/MS and database search Anal Chem 2002 74 20 5383 5392 10.1021/ac025747h 12403597

[B23] Zhang J Gonzalez E Hestilow T Haskins W Huang Y Review of Peak Detection Algorithms in Liquid-Chromatography-Mass Spectrometry Current Genomics 2009 10 6 388 10.2174/138920209789177638 20190954 PMC2766790

[B24] Valkenborg D Assam P Thomas G Krols L Kas K Burzykowski T Using a Poisson approximation to predict the isotopic distribution of sulphur-containing peptides in a peptide-centric proteomic approach Rapid Commun Mass Spectrom 2007 21 20 3387 91 10.1002/rcm.3237 17891751

[B25] Benjamini Y Hochberg Y Controlling the false discovery rate: a practical and powerful approach to multiple testing Journal of the Royal Statistical Society. Series B (Methodological) 1995 57 289 300

