# Supplementary material for: MRCQuant- an accurate LC-MS relative isotopic quantification algorithm on TOF instruments
Source: BMC Bioinformatics. 2011 Mar 15;12:74. doi: 10.1186/1471-2105-12-74 (PMC3072341; doi:10.1186/1471-2105-12-74)
Supplement: Additional file 1 — Support Information. In this file we provide support information. [file 1471-2105-12-74-S1.PDF]

MRCQuant- an accurate LC-MS relative isotopic quantification algorithm on TOF instruments-support information

William E. Haskins[1,2,3,4] , Konstantinos Petritis[5]and Jianqiu Zhang*[6]

[1]Pediatric Biochemistry Laboratory, University of Texas at San Antonio, TX, 78249, USA.
[2]Depts. Biology & Chemistry, University of Texas at San Antonio, TX, 78249, USA.
[3]RCMI Proteomics & Protein Biomarkers Cores, University of Texas at San Antonio, San Antonio, TX 78249, USA.
[4]Dept. of Medicine, Division of Hematology & Medical Oncology, Cancer Therapy & Research Center,University of Texas Health Science Center at San Antonio, San Antonio, TX, 78229, USA.
[5]Center for Proteomics, Translational Genomics Research Institute, Phoenix, AZ 85004, USA.
[6]Dept. Electrical and Computer Engineering, University of Texas at San Antonio, TX 78249, USA.

Email: William E. Haskins - william.haskins@utsa.edu; Konstantinos Petritis - kpetritis@tgen.org; Jianqiu Zhang*- michelle.zhang@utsa.edu;

*Corresponding author

Support Information

To support the mathematical derivations, we first introduce the signal generated by a peptide in LC-MS.

Signals generated by a peptide

A peptide species with molecular weight m_p may generate a group of related peaks in the LC-MS. First, when a peptide species enter the mass spectrometer, different numbers of charges will be attached to them during the ionization process, which results in different charge states. If H^+ is the weight of the charge at charge state z, the resulting m/z value can be calculated as $mz = (m_p + z \times H^+)/z$for $z \in \{1, 2, \cdots\}$. Apart from peptide charge state dispersion, each peptide species should register as a series of isotope peaks in MS. This is due to the fact that different chemical elements that form peptides have isotopes in the natural world. For example, while carbon C^{12} usually has 6 protons and 6 neutrons, it has an isotope with 6 protons and 7 neutrons (C^{13}). The presence of C^{13} will increase the molecular weight of the corresponding peptide species to $m_p + w_c \times iso$, where w_c stands for the weight of the extra neutron and iso is the number of C^{13}s isotopes in the peptide. In summary, for a peptide with mass m_p its m/z value at charge state z and isotope position iso can be calculated as

$$mz_0 = (m_p + w_c * iso + z * H^+)/z \tag{1}$$

Given a total number of peptide counts of a peptide species, the percentage of the peptide composed of iso C^{13} isotopes is governed by the Poisson distribution [1], and is called an isotope pattern. It should be noted that other chemical elements (such as oxygen) may also contribute to the isotope pattern. However, C^{13} is the dominating factor in the formation of an isotope pattern. Also, based on the averaged peptide formula, one can calculated the expected number of carbon atoms based on peptide mass and derive the isotope pattern [1].

The frequency characteristic of LC elution profile variations.

Elution profile variations can be attributed to three sources: Elution process variation before the induction of peptides to the MS instrument, instrument noise, and the so called "Poisson" noise due to the difference between the expected and actual number of peptide isotopes [2]. All three sources will affect the final elution profile observed. Elution process variation is characterized by synchronous up and downs on elution profiles of the same peptide at different isotope positions, since such variation is determined by the interaction between peptides and the gradient in the elution process, which affects all isotopes of the same peptide similarly. The Poisson noise is characterized by synchronous up and downs in opposite directions on most abundant isotope elution profiles. For example, if a peptide has two most significant isotopes at ^{12}C and ^{13}C positions, then given a total number of peptide ions, if the number of ^{12}C isotopes is higher than expected, then the number of ^{13}C isotopes will be lower than expected. Finally, the instrument noise is characterized by unsynchronized noise peaks that show up on different elution profiles randomly. It is quite easy to detect synchronous elution process variations through inspection. Poisson noise is harder to detect in the presence of additive instrument noise. But we know that these to kind of noise changes from scan to scan independently and should share the same frequency characteristics.

We plot a segment of elution profiles of the peptide sequence "HPGDFGADAQGAMTK" in Figure 1. Note that some variations show up synchronously on both ^{13}C and ^{12}C elution profiles, which can be attributed to elution process variations. Some other variations only show up in one profile, which can be attributed to random instrument noise and/or Poisson noise. These variations have the same frequency characteristics in the sense that they have similar peak width.

Determination of MS window

To obtain XICs and MS templates, it is necessary to calculate the m/z values that a peptide signal spans in the m/z dimension. To determine mass window at a given m/z, we utilize the mass resolution

information of the instrument. Here we assume a Gaussian MS peak shape [3]. Suppose the mass resolution is R FWHM and the peak shape is assumed to be Gaussian, then the window size is determined by the following expression:

$$\begin{aligned} dw &= 6\sigma_p \\ &= 6\frac{mz_0}{R\sqrt{\log 4}}, \end{aligned} \tag{2}$$

where σ_p is the standard deviation of the Gaussian shaped MS peak, mz_0 is the mz value of a peptide at a specific charge state and isotope position. mz_0/R calculates the width of the peak at half maximum. Then by further divide it by $\sqrt{\log 4}$, we convert the width to the standard deviation of the Gaussian shaped MS peak. Finally, 6 times the standard deviation is the window size that covers 99% of the peak area.

XIC extraction

At a given m/z value, we first calculate the size of its MS window. The we sum the total ion count within the m/z window in all scans. The resulted XIC is horizontally indexed by scan numbers, and vertically the total ion count in each scan within the MS window.

Extraction of MS template based on MRC

The theoretical derivation of MRC MS template is as the following: During the elution time of a peptide, the peptide registers a copy of its signal in MS scans within the elution time. These copies of peptide signals have the same shape but they are scaled and noise corrupted. We can express the series of peptide signals in the following format. Suppose the signal registered by a peptide in a particular charge state z and isotope position iso can be represented as

$$s_p(mz, t) = A_p(z, iso)lc(t)f(mz - mz_0) + n(mz, t), \tag{3}$$

for $mz \in \mathbf{W}$, where $\mathbf{W} = [mz_1 \cdots mz_N]$ represents all m/z sampling points within the window of the MS peak; $f(mz)$ is the spreading function of the MS instrument; mz_0 is the peptide MS peak central location whose value is calculated using (1) given peptide mass m_p, charge state z and iso. In MS, the peptide signal is not only registered at mz_0, since the MS instrument spread the signal. The MS signal $s_p(mz, t)$ spans the window whose width is determined by the width of $f(mz)$, which can be calculated from the resolution of the MS instrument. $A_p(z, iso)$ is the total count of the peptide ions at a given charge state and isotope position. $lc(t)$ is the fraction of the peptide that elutes out at MS sampling time t. $n(mz, t)$

stands for instrument noise which is assumed to be iid Gaussian. If instrument resolution is high and the occurrence of overlapping MS peaks is rare, then this model is sufficient. Inspecting (3), we find that each $s_p(mz,t)$ at t is a noise corrupted version of $f(mz-mz_0)$ scaled by $A_p(z,iso)lc(t)$, and we can obtain the maximum likelihood estimate of $f(mz-mz_0)$ based on $s_p(mz,t)$ at different ts. The maximum likelihood estimation of this linear and Gaussian model can be obtained by performing "Maximum Ratio Combining (MRC)" which can be found in many references such as [4],

$$\tilde{t}(mz) = \sum_t w(t) * s_p(mz,t), \tag{4}$$

where $w(t)$ is the weight to be assigned to the observation at elution time t, and it should be proportional to the square root of the SNR of the observation. The SNR in this case is given by $(A_p(z,iso)lc(t))^2/\sigma_n^2$. Since the instrument noise variance σ_n^2 is constant from one scan to another, the weight is proportional to $(A_p(z,iso)lc(t))$, which can be estimated based on the observed ion count of the signal at each scan in the m/z range of the peptide. We denote $\tilde{t}(mz)$ as the extracted MS signal template. Also, we can see that if the SNR is small, the implied weight is very small and the effect on signal estimation is minimal. In Figure 2, we show an example of estimated weights for MRC (before normalization). In this figure, the peptide peak is shown in 3D within its MS window. The dashed line represents its LC peak intensities which are used as weights in MRC.

Template translation

In MS spectrums, MS peak width varies with m/z locations, and when translating MS templates, the peak width has to be adjusted. Given a MS template $\tilde{t}(mz)$ that centered around m_0, it is translated to a new m/z location m_1 as $\tilde{t}_1(mz)$ based on the following formula:

$$\tilde{t}_1(mz) = \tilde{t}((mz-mz_1) * \frac{mz_0}{mz_1} + mz_0). \tag{5}$$

This equation is derived based on the fact with a constant resolution, peak width scales linearly with m/z.

Interference Removal

When interference signal exists, we have to perform interference removal over the mz window. In the following, we describe the algorithm: We consider that the local MRC scan as the sum of the MS template signal and the interference signal plus noise

$$\begin{aligned} y(mz) &= a * \tilde{t}(mz) + i(mz) + n \\ &= a * \tilde{t}(mz) + b_0 + b_1 * mz + ... + b_l * mz^l + n \end{aligned} \tag{6}$$

where a is the scale for the template signal. The interference signal $i(mz)$ is approximated as an order l polynomial. $mz \in [mz_0 - dw/2, mz_0 + dw/2$. The above model can also be written in the vector form:

$$\mathbf{Y} = \mathbf{H}\boldsymbol{\theta} + \mathbf{N} \tag{7}$$

where $\mathbf{Y} = [y(mz_1) \cdots (mz_N)]^T$ are all observed MS signal values within the MS window.
$\mathbf{H} = [\mathbf{T}\mathbf{W}^0\mathbf{W} \cdots \mathbf{W}^l]$, where $\mathbf{T} = [\tilde{t}(mz_1)...\tilde{t}(mz_N)]^T$ is the vector of template signals. $\mathbf{W}$ is the vector of mz values. $\mathbf{W}^l = [mz_1^l \cdots mz)_N^l]$ represents the lth order exponential of mz values. $\boldsymbol{\theta} = [a\ b_0\ b_1 \cdots b_l]^T$ is the parameter vector. $\mathbf{N}$ stands for the noise vector. When assuming Gaussian independent noise, the maximum likelihood estimate of the parameters becomes the least square estimate of the parameters. The natural constraint on the parameters is $i(mz) > 0 \forall mz$. Thus, this problem becomes a constrained maximization problem. In addition, since the interference signal could have strong correlations with the signal template, the least square solution could returns an estimate of $i(mz)$ that includes part of $\mathbf{T}$. Thus, when estimating $\boldsymbol{\theta}$, we also need to minimize the correlation between $i(mz)$ and the MS template. This allow us to formulate the problem as a constrained optimization problem with two objectives and one constraint,

$$O_1 : \quad \min_{\boldsymbol{\theta}} (\mathbf{Y} - \mathbf{H}\boldsymbol{\theta})^T (\mathbf{Y} - \mathbf{H}\boldsymbol{\theta}) \tag{8}$$

$$O_2 : \quad \min_{\boldsymbol{\theta}} (a * \mathbf{T}^T * (\mathbf{H} * [0\ b_0\ b_1 \cdots b_l]^T)) \tag{9}$$

$$C_1 : \quad \mathbf{H}[0\ b_0\ b_1 \cdots b_l]^T > 0; \tag{10}$$

We adopted the weighting approach for multiple objective function minimization and the overall objective function is combined as $O_1 + weight * O_2$, where weight is a user defined parameter. Using this formulation, we can utilize the Quadratic Programming [5] to numerically search for the solution of θ. The order of the model l can be selected using the Bayesian Information Criteria (BIC) [6]. It shall be noted that when interference signal is too strong and has significant overlaps with the MS template, the proposed algorithm may not converge. However in general, when the interference signal does not have strong correlation with the MS peptide template, the algorithm converges. In the LC peak detection stage, if interference signal exists and the theoretical template is used, this algorithm returns the estimated height of the peptide signal. If the algorithm does not converge in a scan when strong interference exists, the scan will be excluded from the LC peak. This introduces some error, but the result will be far more accurate than including the scan with strong interference. After LC peak detection, in the quantification stage, this algorithm is applied again to estimate the MS peptide signal height in the MRC combined MS scan. The

MRC combined MS scan have a much higher SNR then any individual MS scan, and in general, the interference signal will also be suppressed significantly. Quantification based on MRC combined scan will significantly improve the accuracy.

References

1. Bayne C, Smith D: **A new method for estimating isotopic ratios from pulse-counting mass spectrometric data**. *International Journal of Mass Spectrometry and Ion Processes* 1984, **59**(3):315–323.
2. Du P, Stolovitzky G, Horvatovich P, Bischoff R, Lim J, Suits F: **A noise model for mass spectrometry based proteomics**. *Bioinformatics* 2008, **24**(8):1070.
3. Coombes K, Koomen J, Baggerly K, Morris J, Kobayashi R: **Understanding the characteristics of mass spectrometry data through the use of simulation**. *Cancer Informatics* 2005, **1**:41–52.
4. Goldsmith A: *Wireless communications.* Cambridge Univ Pr 2005.
5. Fletcher R: **Practical Methods of Optimization: Vol. 2: Constrained Optimization.** *JOHN WILEY & SONS, INC., ONE WILEY DR., SOMERSET, N. J. 08873, 1981, 224* 1981.
6. McQuarrie A, Tsai C: *Regression and time series model selection.* World Scientific Pub Co Inc 1998.

Figure 1 - Elution profile noise characteristics.

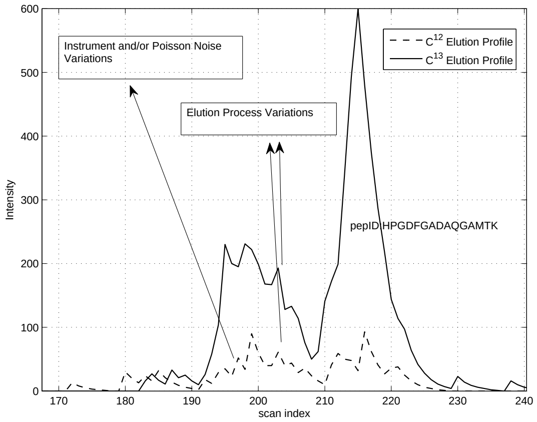

Figure 1: Elution profile noise characteristics. Some variations show up synchronously on both ^{13}C and ^{12}C elution profiles, which can be attributed to elution process variations. Some other variations only show up in one profile, which can be attributed to random instrument noise and/or Poisson noise. These variations have the same frequency characteristics in the sense that they have similar peak width.

Figure 2 - Estimated weights for calculating (4).

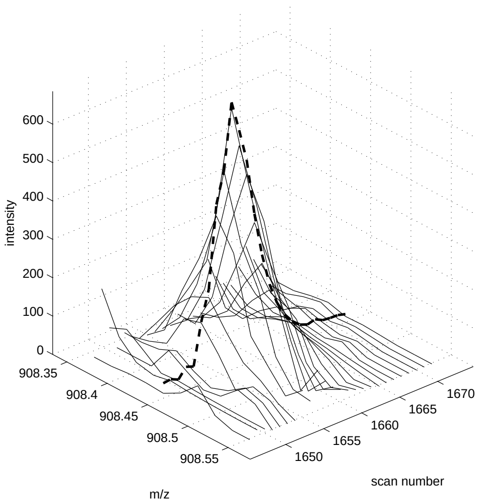

Figure 2: Estimated Weights for Calculating (4) .
